# College and Hospital Notes

**Published:** 1901-03

**Authors:** 


### COLLEGE AND HOSPITAL NOTES.

Brigham Hall, a hospital for mental and nervous diseases, located at Canandaigua, N. Y., is one of the most successful and best managed private institutions in the country for the treatment of mental and nervous diseases. It was established in 1855 by Dr. George Cook, who associated with himself Dr. John B. Chapin, and it is now in charge of Dr. D. R. Burrell. It will be observed, therefore, that it has commanded from its beginning the ablest medical talent in this department of medicine. The buildings, which are of the best class, are beautifully located in one of the most charming regions of the state—a commonwealth abounding in attractive scenery. A handsome booklet is issued by the hospital, which is profusely illustrated.

The University of Pennsylvania has recently issued the Provost's report for the year ending August 31, 1900. It constitutes University Bulletin, No. 2, New Series, contains 250 standard octavo pages and is replete with valuable information. It is a document of interest to educators and bespeaks the progress making in all departments of the great University.

The Charity Eye, Ear and Throat Hospital, located at 166 Broadway, Buffalo, has recently sent out its ninth annual report. During the year ending September 30, 1900, 1983 new cases were treated and 9,671 visits of patients were made to the dispensary, while 21 were received into the hospital. The total cost of maintenance was about $1,900--a most economic exhibition for the amount of excellent work performed.

The Laboratory of Pathology of the University of Buffalo has recently published a report covering the year 1900, which is entitled, Bulletin No. 1. It consists for the most part of reprints of papers published in different medical journals by the laboratory workers. The bulletin has been prepared under the direction of Dr. Herbert U. Williams, director of the laboratory. It contains numerous excellent illustrations, besides material of great interest.

